# Child with temporal lobe hamartoma: A to Z images and a case report

**DOI:** 10.3332/ecancer.2014.436

**Published:** 2014-06-16

**Authors:** José Liders Burgos Zuleta, Roger Carillo Mezo, Eduardo Perusquia Ortega, Beatriz Luna Barrón, Rubén Conde Espinosa, Diana P Marín Muentes, Julián Sánchez Cortázar, María de Guadalupe Gómez Pérez, José Alvaro Burgos Zuleta, José Andres Burgos Zuleta

**Affiliations:** 1 Magnetic Resonance Department, Advanced Medical Image Centre, La Paz, Bolivia; 2 Magnetic Resonance Department, Hospital Ángeles del Pedregal, México, DF 10700, México; 3 Neurology Department, Hospital Ángeles del Pedregal, México, DF 10700, México; 4 Department of Medical Genetics, Universidad Mayor de San Andrés, La Paz, Bolivia; 5 Surgery Department, Hospital Ángeles del Pedregal, Mexico, DF 10700, México

**Keywords:** temporal hamartoma, gelastic seizure, no motivated laughter, magnetic resonance

## Abstract

Gelastic seizure was first described by Trousseau in 1877 and comes from the Greek word gelos (laughs), as laughter is the main feature [1]. Normal laughter is a reactive emotional behaviour and motor action that involves the limbic system, hypothalamus, temporal cortex, and several regions of the brainstem. A female patient, six years old, left-handed, with gelastic seizures, uncontrolled despite being treated with two antiepileptic drugs at high doses, was treated. A simple axial tomography was done, where a hypodense lesion that shapes the inner table of the skull temporal level was observed; later, magnetic resonance imaging was requested, better characterising an intraxial lesion in the right second temporal gyrus cystic appearance.

## Introduction

A hamartoma is a benign focal lesion of normal tissue growing in a disorganised manner anywhere in the body; it may alter the surrounding tissue or not show any symptoms. In this case, it emerged in the temporal region and provoked gelastic seizures (Greek: ‘Gelos’ or ‘laughter’), nervous breakdowns that were first described by Trousseau in 1877 [1].

Remember that normal laughter is an emotional reactive behaviour and a motivated motor action that involves the limbic system, hypothalamus, the temporal cortex, and several regions of the brain stem [1].

These gelastic nervous breakdowns are not a response to motivated actions and go unnoticed by patients who are classified as ‘poorly educated’ and even ‘crazy’. They occur most often in the temporal rather than the front lobe and have also been described as precocious puberty and developmental delay in a study conducted by Tassinari [3].

It is most frequently diagnosed during childhood and the majority of patients will develop intractable epilepsy or other types of seizures, such as complex partial, generalised tonic–clonic seizures, in addition to important psychiatric comorbidity [2].

The development of new image diagnostic methods, such as magnetic resonance imaging (MRI) with spectroscopy or diffusion tensor (DTI) and positron emission tomography (PET), has supplemented the traditional methods, such as computer tomography, to better target treatment decisions and improve the prognosis of these patients.

In general, treatment with medication only does not have good results. Surgical treatment combined with drugs has better results [3], although other authors propose neuronal disconnection in cases of refractory epilepsy or stereotasic endoscopy in cases of endo or periventricular hypothalamic hamartoma [5].

## Presentation of the case

**Figure 1.**
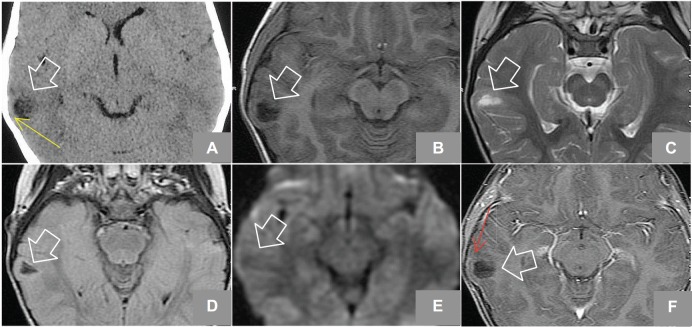
(A) Axial Non enhanced CT shows hypodense lesion in right temporal region, lobulated that shapes the adjacent bone tissue (yellow arrow). MRI axial sequences of: (B) T1w, (C) T2w, (D) FLAIR (Fluid-Attenuated Inversion Recovery), (E) diffusion weighted, and (F) T1w + gadolinium which identifies an image (white arrows) hyperintense on T2w, hypointense on T1w, and FLAIR and does not restrict diffusion w; it is located at the level of the second right temporal gyrus in its portion with discrete anterior subcortical gliosis and lateral enhancement after gadolinium administration (red arrow).

The patient was a female, six years old, left-handed, with gelastic seizures, uncontrolled despite being treated with two antiepileptic drugs at high doses. A simple axial tomography was performed on her and a hypo-dense lesion that shapes the temporal internal table was observed, subsequent to this, a nuclear magnetic resonance was ordered for better characterising of a lesion in the second right temporal gyrus with a cystic appearance (see Figure 1).

**Figure 2.**
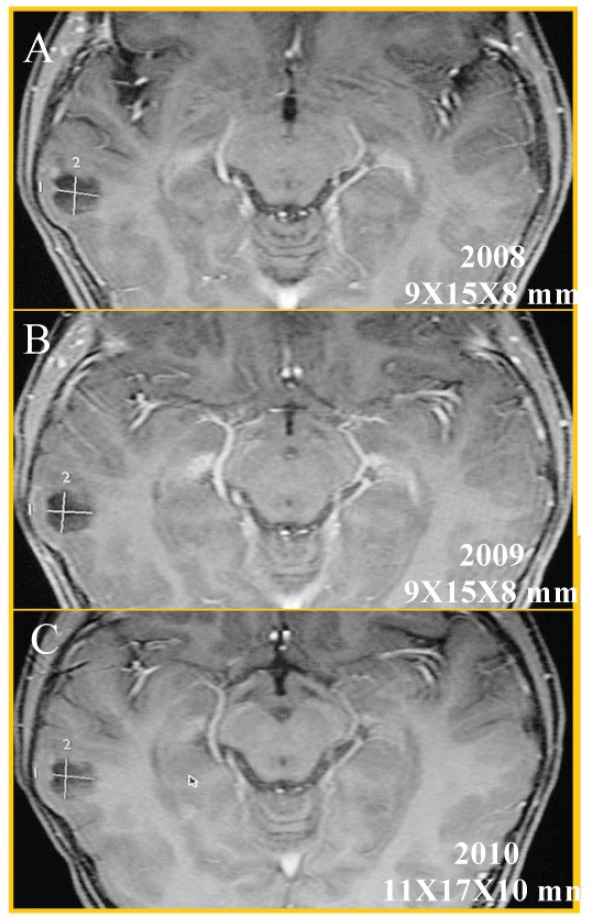
Axial T1w MRI + gadolinium, follow-up of two and a half years; (A) 2008, (B) 2009, and (C) 2010, which showed the temporal injury (white measurements) had grown 2 mm. This lesion measured 9 × 15 × 8 mm and currently measures 11 × 17 × 10 mm along its longest axis.

**Figure 3.**
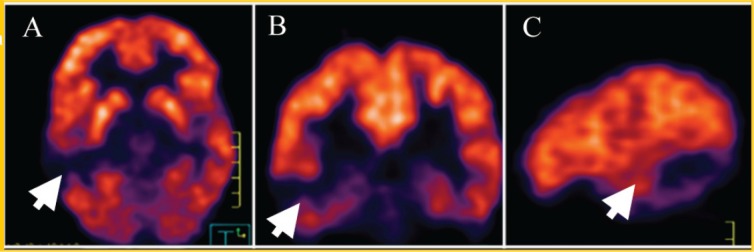
PET (5-FDG) in (A) axial (B) coronal, and (C) sagittal projections that show no uptake in the injury at the right temporal level (white arrow).

**Figure 4.**
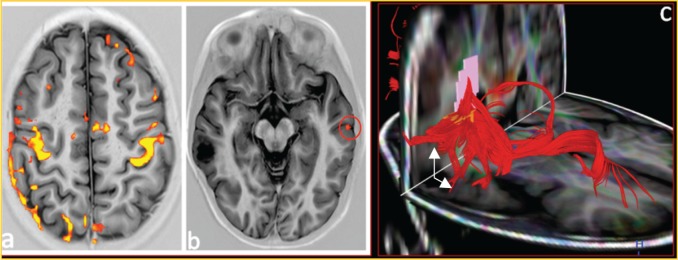
(a) Functional BOLD–MRI shows areas of motor cortex and somato-sensory activation during the bilateral motor palmar paradigm. (b) Areas of activation for the first left temporal girus with participation of the ipsilateral frontopercular region during the paradigm of processing words in silence observing areas of activation in the contra-lateral hemisphere (red circle). (C) 32 directions tractography, shows association fibres of association, demonstrating moved fascicle arcuate (white arrows).

**Figure 5.**
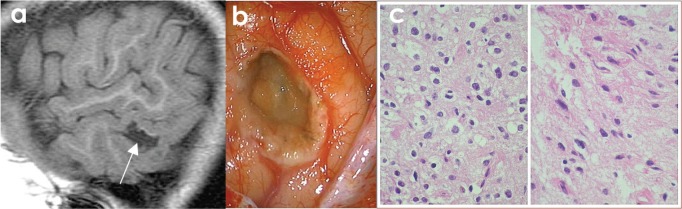
(A) SAGITTAL T1w MRI + contrast: temporal lesion (white arrow). (B) Transoperatory photography (50×) that shows cystic grey lesion and whitish edges. (C) Microscopic photograph (600×) US that shows uniform core astrocytes (70%) immersed in a fibrillar matrix, as well as compact nests of oligodendrocytes (30%) with clear perinuclear halos and a capillary mesh between them, compatible with hamartoma.

This lesion grew slowly (about 2 mm) in the last six months (see Figure 2); because of this, a PET was carried out (see Figure 3) to see the behaviour of the injury; there is no uptake of radiopharmaceutical (5-deoxy Fluoride glucose). Due to the fact that the seizures could not be controlled with medication alone and the increase in the lesion of 2 mm in diameter, a functional magnetic resonance blood-oxygen-level dependence (BOLD) was requested to determine the somato sensory and motor hemispheric dominance, as well as the DTI with 32 directions to define the degree of injury of the white matter tracts, in particular, the tract associated with the speech centre (see Figure 4). This systematic analysis allowed us to decide on the surgical treatment where the benign nature of this lesion is confirmed by histopathology (see Figure 5).

## Discussion

Although this was a single case, the following data pointed more towards a benign than malignant diagnosis: temporal diploe remodelation adjacent to the lesion, cystic appearance, absence of oedema, minimum peripheral enhancement with gadolinium, slow growth (2 mm in the last six months), displacement of association arcuate fibres, and lack of radiopharmaceutical uptake.

In addition, it should be noted that the growth of the injury forced us to determine bilateral activation of motor and somato-sensory areas as well as the dominance of the language area that was left, a finding that allowed an adequate surgical resection without complications or permanent neurologic damage, especially in the case of an infant.

Even though gelastic seizures have been found without a specific clinical pattern, laughter and joy are two clinical elements that involve different and complex mechanisms; therefore, the mere presence of this type of crisis compels us to consider the possibility of a temporal hamartoma or less frequently a frontal hamartoma, without being able to screen out a low-grade glioma that grows faster, has oedema, and more importantly, it is clear that due to the high sensitivity of the histopathological study, this method is definitive.

## Conclusion

**Figure 6.**
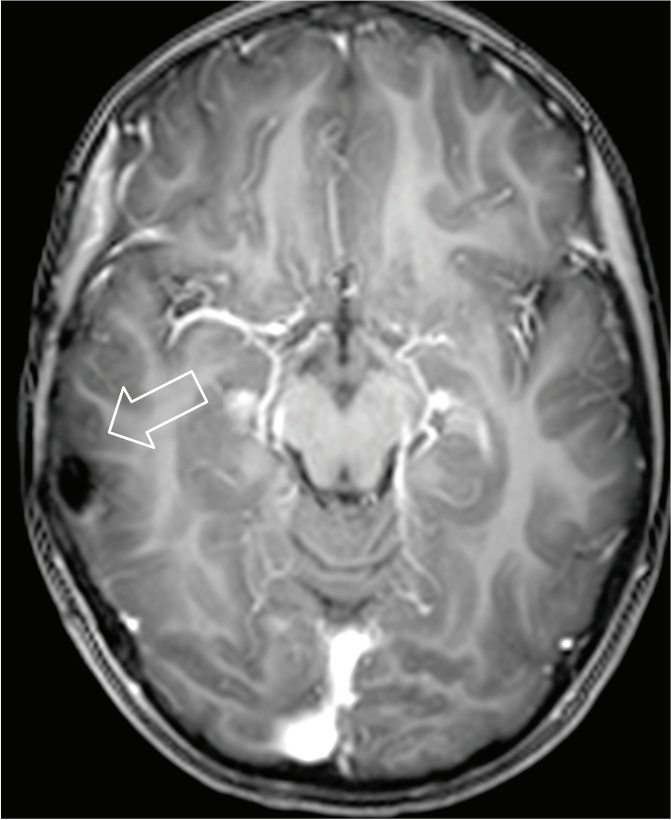
Axial MRI T1w + contrast. 2013 postoperative control. There are post-surgery changes and no evidence of residual lesion or abnormal enhancement.

Temporal hamartoma is a rare event that occurs more frequently in children, therefore we feel that the image method helped us to follow up the injury until measurement modification and determine the dominant hemisphere with functional MRI for surgery without permanent neuronal damage (Figure 6) and with subsequent total control of the seizures with a single anticonvulsant.
